# Cysticlean® a highly pac standardized content in the prevention of recurrent urinary tract infections: an observational, prospective cohort study

**DOI:** 10.1186/1471-2490-13-28

**Published:** 2013-06-05

**Authors:** Francisco Sánchez Ballester, Vicente Ruiz Vidal, Emilio López Alcina, Cristina Domenech Perez, Eva Escudero Fontano, Ana María Oltra Benavent, Ana Montoliu García, Marco Andrés Sobrón Bustamante

**Affiliations:** 1 Urodynamics Unit, Hospital Quirón, Avenida de Vicente Blasco Ibañez 14, 46010, Valencia, Spain; 2 Urology Department, Consorcio Hospital General Universitario, Valencia, Spain; 3 Primary Health Care, Clínica Comarcal Atenea Aldaia, Valencia, Spain; 4 Urology Department Hospital de la Malvarrosa, University of Valencia, Valencia, Spain; 5 Nephrology Department, Hospital Lluis Alcanyis de Xativa, Valencia, Spain

**Keywords:** Cranberry, Cysticlean®, Cystitis, Postcoital, QoL, Urinary tract infections

## Abstract

**Background:**

The present study was aimed at determining the prophylactic efficacy of American cranberry (AC) extract (Cysticlean®) in women with recurrent symptomatic postcoital urinary tract infections (PCUTI), non-consumer of AC extract in the past 3 months before inclusion, and to determine changes in their quality of life (QoL).

**Methods:**

This was a single center, observational, prospective study in a total of 20 women (mean age 35.2 years; 50.0% were married). Patients were followed up for 3 and 6 months during treatment.

**Results:**

The number of PCUTIs in the previous 3 months prior to start the treatment with Cysticlean® was 2.8±1.3 and it was reduced to 0.2±0.5 at Month 6 (*P*<0.0001), which represent a 93% improvement. At baseline, the mean score on the VAS scale (range from 0 to 100) for assessing the QoL was 62.4±19.1, increasing to 78.2±12.4 at Month 6 (P=0.0002), which represents a 20% improvement. All patients had an infection with positive urine culture at baseline, after 6 months there were only 3 symptomatic infections (*P*<0.001). The most common bacterium was *Escherichia coli*.

**Conclusions:**

Prophylaxis with American cranberry extract (Cysticlean®) could be an alternative to classical therapies with antibiotics. Further studies are needed to confirm results obtained in this pilot study.

## Background

The high prevalence of urinary tract infections (UTIs) causes a major social and health impact. 37% of women suffer at least one episode of cystitis during their lifetime, of these, 20% recur in the first two months due to poor cure of infection, resistance to antibiotics or poor hygiene habits. In addition, 12% have recurrent cystitis, that is, they suffer more than 2 episodes of cystitis in a year [1].

Cystitis affects predominantly women aged between 20 and 60 years. The most common causes include: alteration of the vaginal flora, cold and humidity, low insertion of the urethral meatus, chronic constipation, lack of lubrication and frequency of sexual intercourse, urinary retention and misuse of antibiotics.

To deal with this situation, different therapeutic options have been proposed. After establishing a presumptive diagnosis of UTI, antibiotic therapy is instituted until the result of the antibiotic susceptibility testing of the urinary culture is obtained. When infections are recurrent, the possibility of administering antibiotic prophylaxis temporarily can be considered, though given the gradual increase in the number of antibiotic resistances of the most common pathogens, the current trend is to search for other options [2].

Cysticlean® (*Vaccinium macrocarpon*) (hereinafter named Cys) is a dietary food supplement based on concentrated American cranberry (AC) extract. AC is known for its antibacterial properties and benefits on health of the urinary tract due to its high content in proantocyanidins (PAC) and, to a lesser extent, quinic acid [3]. There is not a standard method (European Pharmacopeia) to measure PAC contents on AC, and this is the reason AC preparations with similar PAC content do not have the same antiadhesive effect against *Escherichia coli*. Cys is the AC extract marketed in Spain with greater antiadhesive capacity against *E. coli* (83% and 76% in the sachet and capsule forms, respectively), the main cause of UTIs [4].

The use of concentrated AC extract has no reported contraindications or side effects. It is thus ideal as a long-term treatment to prevent cystitis recurrences. Very rare cases of interaction with warfarin have been reported, but all were associated with massive intake of AC juice for several days [2].

Based on the above, this pilot study was conducted to determine the prophylactic efficacy of AC extract (Cys) in women with recurrent symptomatic postcoital urinary tract infections (PCUTIs) and its impact on their quality of life.

## Methods

### Study design and patient selection

**Figure 1:**
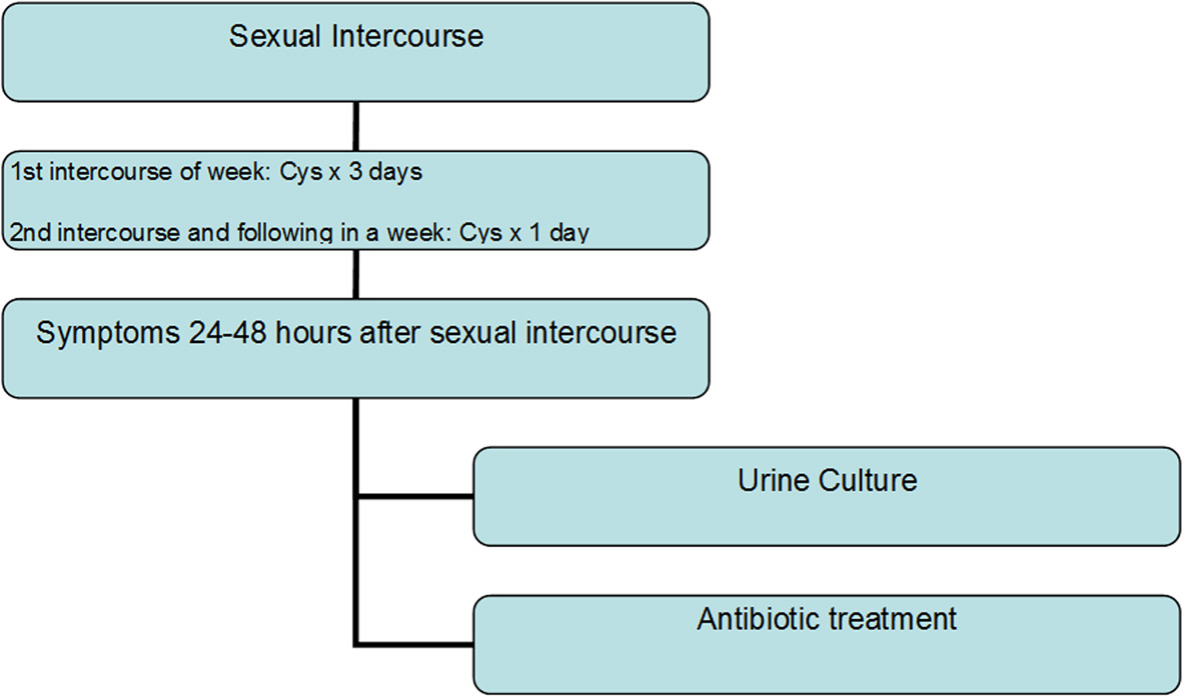
Flow diagram.

This was a single center, observational, prospective study conducted in an Urodynamics Unit (Voiding Function Unit) in Voiding Functional Unit, Hospital Quiron, Valencia, Spain from August 2011 to June 2012. We consider PCUTI when the symptoms appeared up to 48 hours after sexual intercourse [5,6]. The study included women aged 18 to 60 years old, with at least two PCUTIs due to E. coli in the past year and with active infection confirmed by symptomatic positive urine culture to *E. coli* at the beginning of the study. After treatment with antibiotics a culture was taken to confirm the absence of infection. Patients were counseled in previous visits about vaginal hygiene measures, particularly avoiding use of spermicidal creams and oral contraceptives. Patients with regular consumption of Cys within the past three months, a bladder neoplasm, urinary stones, postvoid residual urine > 100 cc, or obstructive urinary symptoms were excluded from the study. Patients included received Cys (PACs 118 mg/day) for 6 months as follows: after 1^st^ sexual intercourse in a week received 1 Cys sachet daily for 3 days, and after 2^nd^ and following intercourses in a week received only 1 Cys sachet postcoital (Figure 1).

The primary objective of the study was to determine the prophylactic efficacy of AC extract in women with PCUTIs. The secondary objectives were to determine the tolerability of Cys, and to determine changes in QoL of patients with PCUTIs taking Cys.

The study was carried out according to the European Guidelines for Good Epidemiological Practice [7], and the provision of the Declaration of Helsinki (as revised in Tokyo 2008). The study was approved by the Clinical Research Ethics Committee of the University of Valencia, and followed the Spanish Law 15/1999 on Personal Character Data Protection concerning confidentiality of patients’ data. All patients participating in the study signed the corresponding written consent form.

### Study variables

The total length of the study was 6 months per patient, including 3 visits (Baseline, Month 3 and Month 6). The following patient data were collected using a Case Record Form specifically designed for this study: sociodemographic data, concomitant diseases, microbiological data, characteristics of PCUTIs, voiding habits, vaginal hygiene, QoL, patient status in the study and adverse events.

Urine culture was carried out for patients presenting at least one symptom of infection and if the result was positive they were administered antibiotic treatment. In this way microbiological data were only obtained in presence of PCUTIs after treatment with Cys.

QoL was measured at each visit asking for wellbeing of patients including sexual relations by using a Visual Analogue Scale (VAS), ranging 0–100 mm, where 0 is the worst imaginable health state and 100 the best imaginable health state.

### Statistical analyses

Descriptive statistics were performed for all variables analyzed. Measures of central tendency and dispersion with their 95% confidence interval are shown for quantitative variables, while absolute and relative frequencies are shown for qualitative variables.

The distribution type of the continuous variables was studied and its adjustment to the Gaussian distribution was assessed using the Kolmogorov-Smirnov test. The statistical significance of the changes from baseline at Month 3 and Month 6 (analysis of the paired data) was obtained using Student’s t-test for quantitative variables, the McNemar test for dichotomous qualitative variables, and the McNemar-Bowker test for ordinal qualitative variables. *P*-values were based on two-sided testing at a 5% significance level. All data were analyzed using SAS statistical package version 9.0.

## Results

### Sociodemographic data and concomitant diseases

The mean age of the 20 patients included in the study was 35.2 (± 9.6) years, all had been schooled and 50.0% were married. Only 5 patients had concomitant diseases: 2 (10.0%) had dyslipemia, 1 (5.0%) had multiple sclerosis, 1 (5.0%) had hypertension and 1 (5.0%) had allergic rhinitis.

### Clinical data

The mean number of PCUTIs over the previous 3 months at baseline (prior to start treatment with Cys) significantly decreased from 2.8 (± 1.3) at baseline to 0.7 (± 1.0) at Month 3 (P<0.0001) and continued decreasing to 0.2 (± 0.5) at Month 6 (P<0.0001). Table 1 shows the percentage of patients with PCUTIs.

**Table 1:** Percentage of patients with PCUTIs at each visit

	**Baseline (prior to start of treatment)**	**Month 3**	**Month 6**
	**(n=20)**	**(n=20)**	**(n=20)**
Percentage of patients with PCUTIs (previous 3 months)	20 (100%)	10 (50 %)	3 (15 %)
P-value vs. Baseline		0.0016*	<0.0001*
P-value month 6 vs. Month 3			0.0082*

* statistically significant.

At inclusion, patients reported that they had presented chronic PCUTIs for 4.3 ± 2.6 years on average.

### Voiding habits

Concerning voiding habits over the previous 3 months, at baseline 40.0% of patients reported urinating before intercourse and 65.0% reported urinating after intercourse, and after 3 months these percentages increased to 50.0% and 75.0%, respectively, being maintained after 6 months. 40.0% of patients reported being regular urinators at baseline as well as at Month 6. No statistically significant differences were found in any case, except for tendency to retain urine reported by 25.0% of patients at baseline and significantly decreasing (*P*<0.05) to 5.0% both at Month 3 and at Month 6.

### Vaginal hygiene

There were no changes in vaginal hygiene from baseline visit during the study. 70.0% of patients practiced normal vaginal hygiene (once daily with daily bath, without use of antiseptic substances or special soaps), 25.0% practiced moderate hygiene (once or twice daily, postcoital or after urinating), and 5.0% practiced extreme hygiene (several times a day and even with vaginal antiseptic).

### Partners, spermicidal creams and oral contraceptives

Patients did not report change in the kind and frequency of intercourse, maintained the same partner and reported no changes in use of spermicidal creams and oral contraceptives.

### QoL (VAS)

The mean change from baseline on the VAS scale (range from 0 to 100) was 8.7 (±13.7) at Month 3 and 15.8 (± 15.5) at Month 6. Both changes were statistically significant (*P*=0.0110 and *P*=0.0002, respectively).

**Figure 2:**
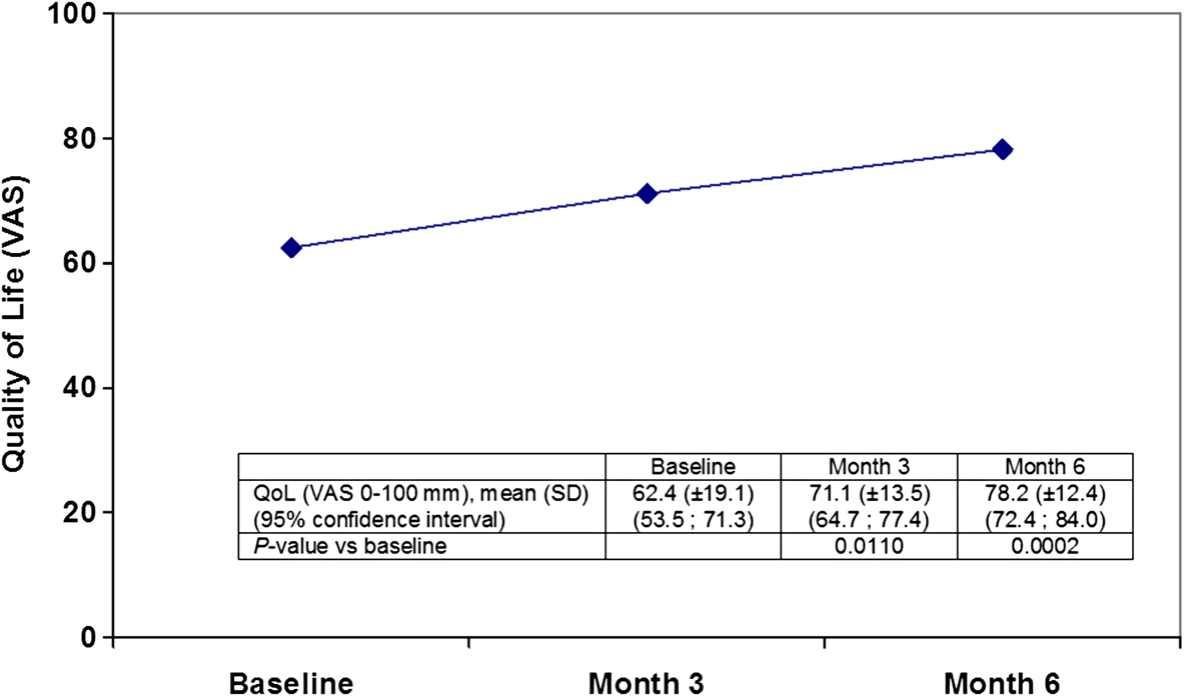
**Quality of life (VAS).** Results are expressed as mean (± SD) with the corresponding 95% confidence intervals.

Figure 2 shows the mean score per visit with the corresponding 95% confidence intervals.

### Presence of bacteria causing PCUTIs

As per the inclusion criteria, at baseline all 20 patients had an infection with positive urine culture to the presence of bacteria causing PCUTIs (Table 1).

The most common bacteria causing PCUTIs was *E. coli*, being present in all 20 patients at baseline and significantly decreasing its presence to 6 patients after 3 months (*P*=0.0002) and to only 2 patients after 6 months (*P*<0.0001).

### Adverse events

No adverse events occurred during the study and no patient had side effects derived from taking AC extract.

## Discussion and conclusion

As far as we know this is the first study conducted to directly assess the potential benefit of AC extracts in the prevention of PCUTIs. Recently published reviews on urinary tract infections management include the use of AC products, but none specifically address postcoital UTI [8-11]. In a systematic review of literature carried out on the use of AC in chronic urinary infections, no studies on postcoital use of AC were found [12].

AC is a widely used and recommended remedy for prophylaxis of UTI and several clinical trials have documented its efficacy in women with recurrent UTI [4,13,14]. AC contains type A proanthocyanidin (PAC) that inhibits adherence, in doing so preventing the colonization of the *E. coli* uropathogen in the vaginal mucosa and reducing the frequency of bacteriuria, and this effect occurs in a dose dependent manner [15-19].

According to a recent updated Cochrane review [20], AC juice does not appear to have a significant benefit in preventing UTI, even though on a prior update it appeared that there was some evidence that AC juice may decrease the number of symptomatic UTIs over a 12 month period, particularly for women with recurrent UTIs [21]. However, the update recommended more studies to evaluate AC products such as tablets or capsules on women with recurrent UTIs (20). In a randomized controlled trial in elderly women to compare the efficacy in preventing UTI of AC capsules versus trimethoprim in preventing UTI, there were no significantly differences between groups concerning time to first recurrence of UTI, and trimethoprim had a very limited advantage over AC in the prevention of recurrent UTI in older women, but had more adverse effects and withdrawals [22]. In the elderly population there was only one clinical trial with asymptomatic bacteriuria, which showed that bacteriuria and pyuria were significantly reduced in women taking AC juice in comparison with women who received placebo [23]. Several meta-analyses have established that recurrence rates over 1 year are reduced approximately 35% in young to middle-aged women [24].

The results of the present study confirmed the efficacy of Cys as previously described, with a significant decrease of 75% in the number of PCUTIs after 3 months of Cys intake, until 93% after 6 months. Although the composition of effective AC products and its dosage in UTI prophylaxis have not yet been defined, 13 it has been demonstrated that AC extract containing higher doses of PACs produce a greater benefit [25] and a dose of 118 mg/day of PACs has demonstrated a significant efficacy in different clinical trials with Cys [26].

The current management of recurrent UTI involves repeated courses of antibiotics or subtherapeutic, long-term antibiotic prophylaxis [27]. However, adverse effects are common with these treatments, as well as increasing antibiotic resistance [28]. The increasing prevalence of *E. coli* isolates (the most common uropathogen) that are resistant to antimicrobial agents, together with the high costs of antibiotic treatments, have stimulated interest in novel non-antibiotic methods for the prevention of UTI [25]. Given the reduction of the number of antibiotic treatments per patient with the use of Cys, as demonstrated by the results of the present study, this should theoretically decrease occurrence of microbial resistance.

Prevention of recurrent UTIs includes early postcoital voiding habits [29,30]. In fact, the American College of Obstetricians and Gynecologists District II NYS recommends urinating after sexual intercourse to prevent recurrent cystitis. Nevertheless, there is no proven association between recurrent UTIs and pre or postcoital voiding patterns, frequency of urination, wiping patterns, douching, use of tight undergarments, or delayed voiding habits [31,32]. The results from the present study showed increasing urinating habits before and after sexual intercourse of approximately 20% already after 3 months of AC extract daily intake. Furthermore, as there were no changes in vaginal hygiene during the study, the improvement seen in PCUTIs was mainly attributable to Cys daily intake.

One issue for discussion is the pattern of treatment to be administered. We decided a treatment of 3 days simulating the guidelines of antibiotic treatment in PCUTI [33]. However, based on the dose dependent effect of PAC and previous experience with antibiotic profilaxis in PCUTI, we suspect that the administration of higher doses in a shorter interval of time should be equally effective.

Urinary tract infections have an detrimental impact on patients QoL [34,35]. Although uncomplicated UTI in women is considered to be a relatively benign and self-limiting condition, it is responsible for significant symptomatology, morbidity and loss of QoL and causes unnecessary suffering, affecting patient’s daily activities, social functioning and wellbeing, and sexual intercourse which, is not satisfactory [36]. Consequently, a decrease of recurrent symptomatic infections should relate to an increase of QoL. In the present study we use a VAS score to assess QoL. Although not a standardized method for assessing QoL, we selected it for its simple completion and interpretation. There was a significant increase of 20% in the QoL evaluation related to the decrease found in the mean number of PCUTIs in which patients had taken antibiotics after 6 months of Cys daily intake.

Finally, in the present study there was a significant reduction of patients with symptomatic infections after 3 months and again after 6 months. It should be taken into account that urine cultures were only carried out in presence of PCUTIs, therefore the high percentage of women with culture not done should be considered as a treatment success. Thus Cys improves QoL decreasing infections derived from sexual intercourse. Further studies are needed to confirm results obtained in this pilot study.

The use of AC products appears to be safe. The present study confirmed the safe profile of Cys as there were no adverse events at all. Prophylaxis of Cys seems to be a promising option to decrease the number of PCUTIs and therefore increase QoL.

The results of this pilot study are not significant due to the short follow-up period and the limited number of patients. We believe is needed a randomized placebo controlled study with at least 50 patients and follow up should be one year. However, over longer periods of time changes in sexual habits, contraception methods, or regular partner can occur, which may invalidate the study.

## Competing interests

The authors declare that they have no competing interests.

## Authors’ contributions

FSB has made substantial contributions to conception, funding, design, supervision, data collection, interpretation of data and critical revision of the manuscript; ELA has made contributions to data collection, data interpretation and providing a critical review of the manuscript; VRV has made contributions in providing a critical review of the manuscript for important intellectual content; CDP has made contributions to technical support, and provided a critical review of the manuscript; EEF, AOB, AMG and MSB have made contributions providing a critical review of the manuscript for important intellectual content. All authors read and approved the final manuscript.

## Pre-publication history

The pre-publication history for this paper can be accessed here:

http://www.biomedcentral.com/1471-2490/13/28/prepub

## References

[B1] Data available at Spanish Statistical Office (2005) and National Survey of the perception of Cystitis Spanish Society of Gynecology and Obstetrics and Spanish Association of Urology 2007

[B2] Rousaud Baron A Urinary tract infection and cystitis. The contribution of Phytotherapy. The red cranberry Urol Integ Invest 2007

[B3] http://www.cysticlean.com

[B4] Risco E Miguélez C Sánchez de Badajoz E Rouseaud A Effect of American cranberry (Cysticlean) on Escherichia coli adherence to bladder epithelial cells. In vitro and in vivo study Arch Esp Urol 2010 63 422 430 20820081

[B5] Neinstein LS Baltimore M Genitourinary tract infections Adolescent Health Care A Practical Guide 1996 3 Baltimore, Md: Williams & Wilkins 431 444

[B6] Stapleton A Latham RH Johnson C Stamm WE Postcoital antimicrobial prophylaxis for recurrent urinary tract infection. A randomized, double-blind, placebo-controlled trial JAMA 1990 264 703 706 10.1001/jama.1990.03450060049027 2197450

[B7] European Medicines Agency (EMEA) Note for guidance on Good Clinical Practice CPMP/ICH/135/95 2002

[B8] Sen A Recurrent cystitis in non-pregnant women Clin Evid (Online) 2008

[B9] Kodner CM Thomas Gupton EK Recurrent urinary tract infections in women: diagnosis and management Am Fam Physician 2010 82 638 43 20842992

[B10] Wagenlehner FM Vahlensieck W Bauer HW Weidner W Naber KG Piechota HJ Primary and secondary prevention of urinary tract infections Urologe 2011 50 1248 56 10.1007/s00120-011-2616-5 21927878

[B11] Nosseir SB Lind LR Winkler HA Recurrent uncomplicated urinary tract infections in women: a review J Womens Health (Larchmt) 2012 21 347 54 10.1089/jwh.2011.3056 22136339

[B12] Bruyère F Use of cranberry in chronic urinary tract infections Med Mal Infect 2006 36 358 63 10.1016/j.medmal.2006.05.001 16857334

[B13] Nowack R Schmitt W Cranberry juice for prophylaxis of urinary tract infections - Conclusions from clinical experience and research Phytomedicine 2008 9 653 67 18691859 10.1016/j.phymed.2008.07.009

[B14] Howell AB Bioactive compounds in cranberries and their role in prevention of urinary tract infections Mol Nut Food Res 2007 51 732 7 10.1002/mnfr.200700038 17487930

[B15] Raz R Urinary tract infection in elderly women Int J Antimicr Agents 1998 10 177 9 10.1016/S0924-8579(98)00024-7 9832278

[B16] Raz R Urinary tract infection in postmenopausal women Korean J Urol 2011 52 801 8 10.4111/kju.2011.52.12.801 22216390 PMC3246510

[B17] Howell AB Vorsa N Marderosian AD Foo LY Inhibition of the adherence of P-fimbriated Escherichia coli to uroepithelia-cell surfaces by proanthocyanidins extracts from cranberries N Engl J Med 1998 339 1085 6 10.1056/NEJM199810083391516 9767006

[B18] Di Martino P Agniel R David K Reduction of Escherichia coli adherence to uroepithelial bladder cells after consumption of cranberry juice: a double-blind randomized placebo-controlled cross-over trial World J Urol 2006 24 21 7 10.1007/s00345-005-0045-z 16397814

[B19] Gupta K Chou MY Howell A Wobbe C Grady R Stapleton AE Cranberry products inhibit adherence of p-fimbriated Escherichia coli to primary cultured bladder and vaginal epithelial cells J Urol 2007 177 2357 60 10.1016/j.juro.2007.01.114 17509358 PMC3684265

[B20] Jepson RG Williams G Craig JC Cranberries for preventing urinary tract infections Cochrane Database Syst Rev 2012 10 CD001321 23076891 10.1002/14651858.CD001321.pub5 PMC7027998

[B21] Jepson RG Craig JC Cranberries for preventing urinary tract infections Cochrane Database Syst Rev 2008 1 CD001321 18253990 10.1002/14651858.CD001321.pub4

[B22] McMurdo ME Argo I Phillips G Daly F Davey P Cranberry or trimethoprim for the prevention of recurrent urinary tract infections? A randomized controlled trial in older women J Antimicrob Chemother 2009 63 389 95 19042940 10.1093/jac/dkn489 PMC2639265

[B23] Avorn J Monane M Gurwitz JH Glyn RJ Choodnovsky I Lipsitz LA Reduction of bacteriuria and pyuria after ingestion of cranberry juice JAMA 1994 271 751 4 10.1001/jama.1994.03510340041031 8093138

[B24] Guay DR Cranberry and urinary tract infections Drugs 2009 69 775 807 10.2165/00003495-200969070-00002 19441868

[B25] Howell AB Botto H Combescure C Dosage effect on uropathogenic Escherichia coli anti-adhesion activity in urine following consumption of cranberry powder standardized for proanthocyanidins content: a multicentric randomized double-blind study BMC Infect Dis 2010 10 94 10.1186/1471-2334-10-94 20398248 PMC2873556

[B26] Rousaud A American cranberry (Cysticlean®) and its use in the prevention of urinary tract infections Monography 2012

[B27] Hooton TM Recurrent urinary tract infection in women Int J Antimicrob Agents 2001 17 259 68 10.1016/S0924-8579(00)00350-2 11295405

[B28] Hisano M Bruschini H Nicodemo AC Srougi M Cranberries and lower urinary tract infection prevention Clinics 2012 67 661 7 10.6061/clinics/2012(06)18 22760907 PMC3370320

[B29] Nickel JC Practical Management of recurrent urinary tract infections in premenopausal women Rev Urol 2005 7 11 7 16985802 PMC1477561

[B30] Foxman B Frerichs RR Epidemiology of urinary tract infection: II. Diet, clothing, and urination habits Am J Public Health 1985 75 1314 7 10.2105/AJPH.75.11.1314 4051067 PMC1646695

[B31] Hooton TM Recurrent urinary tract infection in women Int J Antimicrob Agents 2001 17 4 259 268 10.1016/S0924-8579(00)00350-2 11295405

[B32] Scholes D Risk factors for recurrent urinary tract infection in young women J Infect Dis 2000 182 4 1177 1182 10.1086/315827 10979915

[B33] Albert X Huertas I Pereiró II Sanfélix J Gosalbes V Perrota C Antibiotics for preventing recurrent urinary tract infection in non-pregnant women Cochrane Database Syst Rev 2004 3 CD001209 15266443 10.1002/14651858.CD001209.pub2 PMC7032641

[B34] Espuña Pons M Puig CM Lower urinary tract symptoms in women and impact on quality of life.Results of the application of the King’s Health Questionnaire Actas Urol Esp 2006 30 684 91 10.1016/S0210-4806(06)73518-5 17058613

[B35] Ellis AK Verma S Quality of life in women with urinary tract infections: is benign disease a misnomer? J Am Board Fam Pract 2000 13 392 7 11117334 10.3122/15572625-13-6-392

[B36] Eriksson I Gustafson Y Fagerström L Olofsson B Do urinary tract infections affect morale among very old women? Health Qual Life Outcomes 2010 8 73 10.1186/1477-7525-8-73 20650004 PMC2920245

